# The First Cathedral on America’s Pacific Coast

**DOI:** 10.1007/s41636-020-00275-z

**Published:** 2021-01-04

**Authors:** Iosvany Hernández Mora, Juan G. Martín, Bethany Aram

**Affiliations:** 1 Department of History and Social Sciences, Universidad del Norte, Km 5 via Puerto Colombia, Barranquilla, Colombia; 2 Estación Científica Coiba AIP, Edificio 205, Oficina 117, Ciudad del Saber, Panama City, Panama; 3 Department of History and Social Sciences, Universidad del Norte, Km 5 via Puerto Colombia, Barranquilla, Colombia; 4 Department of Geography, History and Philosophy, Universidad Pablo de Olavide, Carretera de Utrera, Km 1, 41013 Seville, Spain

**Keywords:** Panamá Viejo, funerary archaeology, foundations, commemoration

## Abstract

New research dispels the idea that Panamá Viejo was initially founded one-half mile from the site of its visible present-day ruins. The archaeological and historical evidence, subjected to interdisciplinary analysis, demonstrates that the city remained on the same main plaza next to its natural port from its founding 500 years ago until its destruction in 1671. The data reconsidered and newly uncovered also suggest reasons for previous misinterpretations of the city’s early foundational history. Unlike many colonial cities and towns, Panamá Viejo did not move during its first century of existence. However, its main church, which became the bishopric’s cathedral in 1524, did relocate after 1541. The new evidence establishes and confirms the original location of the first cathedral on America’s Pacific Ocean to the south of Panamá Viejo’s main plaza and explains its move to an elevated, rocky area on the eastern side of the same plaza over 20 years later. Excavations undertaken in 2018 have confirmed the original building’s location a mere 50 m from the visible ruins of the cathedral, the tower of which remains a symbol of Panamanian identity today.

## Resumen

Las nuevas investigaciones despejan la idea de que Panamá Viejo fue inicialmente fundada a milla y media del sitio en donde ahora son visibles sus ruinas. Las evidencias arqueológicas e históricas, sometidas a un análisis interdisciplinar, demuestran que la ciudad permaneció en el mismo emplazamiento, al lado de su puerto natural, fundada 500 años atrás hasta su destrucción en 1671. La revisión de la información disponible, además de la descubierta recientemente, también sugieren las razones de los errores interpretativos previos en torno a la historia fundacional de la ciudad. A diferencia de numerosas ciudades y pueblos coloniales, Panamá Viejo no fue trasladada durante su primer siglo de existencia. Sin embargo, su iglesia principal, la cual se convirtió en sede catedralicia en 1524, fue reubicada después de 1541. La nueva evidencia confirma la localización original de la primera catedral en el Pacífico americano al sur de la plaza mayor de Panamá Viejo y explica el traslado a un área rocosa más elevada, en el costado este de esta plaza, unos veinte años más tarde. Las excavaciones adelantadas en el 2018 han confirmado su ubicación original a escasos 50 m de las actuales ruinas de la catedral, cuya torre permanece como un símbolo de la identidad panameña hoy.

## Résumé

Une nouvelle recherche écarte l'idée que la fondation de Panama Viejo soit intervenue originellement à 800 m du site de ses ruines visibles de nos jours. Les preuves historiques et archéologiques, lorsqu'elles sont assujetties à une analyse interdisciplinaire, démontrent que la ville est demeurée sur la même place principale que son port naturel à compter de sa fondation il y a 500 ans jusqu'à sa destruction en 1671. Les données ayant fait l'objet d'un nouvel examen et qui ont été découvertes suggèrent également les motifs à l'appui des interprétations erronées de l'histoire de la fondation initiale de la ville. Contrairement à de nombreuses villes et cités coloniales, Panama Viejo ne s'est pas déplacée au cours du premier siècle de son existence. Cependant, son église principale qui est devenue en 1524 la cathédrale de l'évêché, s'est implantée ailleurs après 1541. Les preuves nouvelles établissent et confirment le site originel de la première cathédrale sur l'Océan pacifique de l'Amérique au sud de la place principale de Panama Viejo et expliquent son déplacement vers une zone élevée, rocheuse sur la partie orientale de la même place plus de 20 ans par la suite. Les fouilles entreprises en 2018 ont confirmé le site de la bâtisse d'origine à seulement 50 m des ruines visibles de la cathédrale, dont la tour demeure un symbole de l'identité panaméenne de nos jours.

## Introduction

**Fig. 1:**
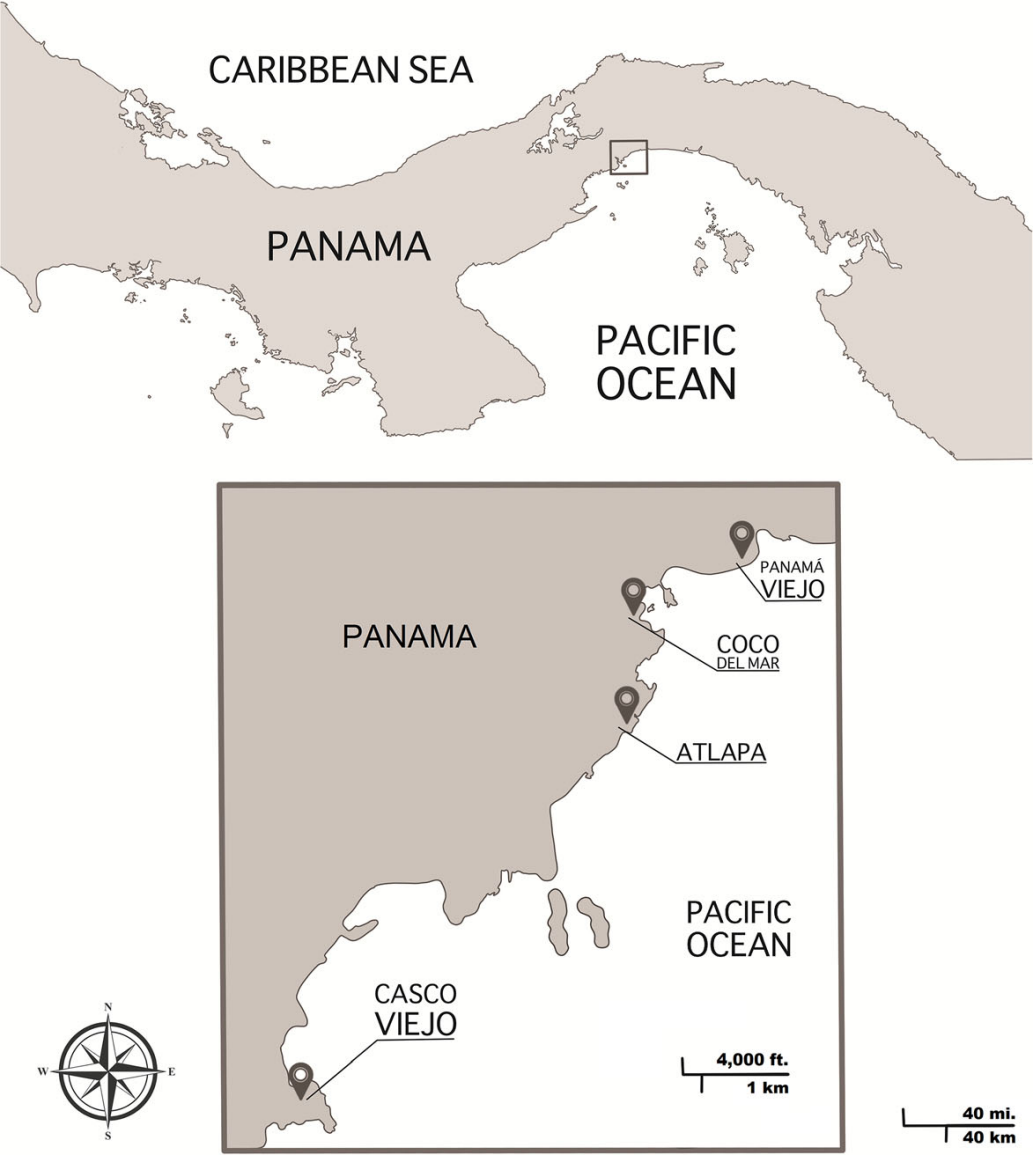
The general location of Panama. Detailed information about Panamá Viejo´s location and nearby areas. (Drawing by Manuela Martín, 2019.)

A number of early Spanish settlements in the Americas were moved shortly after their original founding, including Santo Domingo and the “City of the Kings,” or Lima. In two cases––those of Vera Cruz and Havana––such relocation took place in 1519, the year that also witnessed the settlement of the first European city on America’s Pacific coast, Panamá Viejo (Fig. 1). However, unlike other settlements, the city established by Pedrarias Dávila in 1519 was not moved for over 150 years. The historiography has erroneously considered multiple sites for Panamá Viejo’s original location, based on 17th-century sources situating it either one-half mile or one-half league (1.7 mi.) from today’s identifiable ruins and historical site (Castillero 2006:2, 2017:18). The misinterpretation of such 17th-century sources, with implications for archaeological research (Martín and Rovira 2012) as well as for the construction of memories and identities (Martín and de Arango 2013; Linero 2017), can be revised in light of the new historical and archaeological evidence presented in the following pages.

Conscious of the 500-year commemoration (1519–2019), our interdisciplinary research team has sought to illuminate the city’s founding as well as the lives and deaths of its first European and African residents with attention to the presence of Native Americans, who had inhabited the area since A.D. 600 (Mendizábal 2004). Our interdisciplinary project, funded by the European Research Council (ERC CoG 648535): “An ARTery of Empire,” or “ARTEmpire” for short, considers the impact of early globalization across the Isthmus of Panama through coordinated research in historical archives, such as Seville’s Archivo General de Indias (General Archive of the Indies), and archaeological excavations undertaken in 2017 and 2018 at the Panamá Viejo archaeological site.

The archaeological site of Panamá Viejo covers over 28 protected hectares in Panama City. Since 1995, evidence of pre-Hispanic habitation has been excavated from different areas of the site, including the Plaza Mayor/Casas Oeste (main plaza/western houses), where Tomás Mendizábal (2004) found burials with funerary offerings, and the Parque Morelos/Centro de Visitantes (Morelos Park/visitors’ center), where Juan G. Martín (2002) encountered pre-Hispanic burials and evidence of pre-Hispanic dwellings (post molds). Pre-Hispanic burials have also been found in the nearby residential area of Coco del Mar (Fig. 1). In and around Panamá Viejo’s main plaza, but not in Coco del Mar, early colonial burials have also been found in proximity to the pre-Hispanic ones. The pre-Hispanic burials feature elaborate offerings as well as the reburial of other individuals in ceramic urns or bundles, and, in two cases, the arrangement of male skulls around individual female skeletons. Such was the case in the main plaza, which, on the eve of the conquest, appears to have been a burial ground rather than a site of pre-Hispanic residence, with other nearby areas used for habitation as well as burial grounds. Hence the first 300–400 European settlers who founded Panamá Viejo, with African as well as indigenous slaves and allies, appear to have built the city in proximity to an indigenous settlement, but not on top of it.

Before the 18th century, Roman Catholic burials commonly took place beneath the floors of standing churches rather than in cemeteries (Zucchi 2006; Hernández Domínguez 2013). Normally, architectural evidence establishes the location of such churches. In the absence of architectonic features, however, this article argues that Catholic burials can provide enduring evidence of the earliest colonial contexts. We advance this interpretation, specifically for colonial burials found in an east–west orientation parallel and very close to Panamá Viejo’s present-day coastline some 50 m from the cathedral’s visible ruins and in front of those of the Iglesia de la Merced (Mercedarian church), respectively. Colonial Catholic burials have also been excavated within the 17th-century visible ruins of Panamá Viejo’s churches, including its famous cathedral (1541–1671) and adjacent cemetery, the churches of Santo Domingo (1566–1671), Nuestra Señora de la Concepción (1594–1671), and the Hospital de San Juan de Dios (1620–1671).

**Fig. 2:**
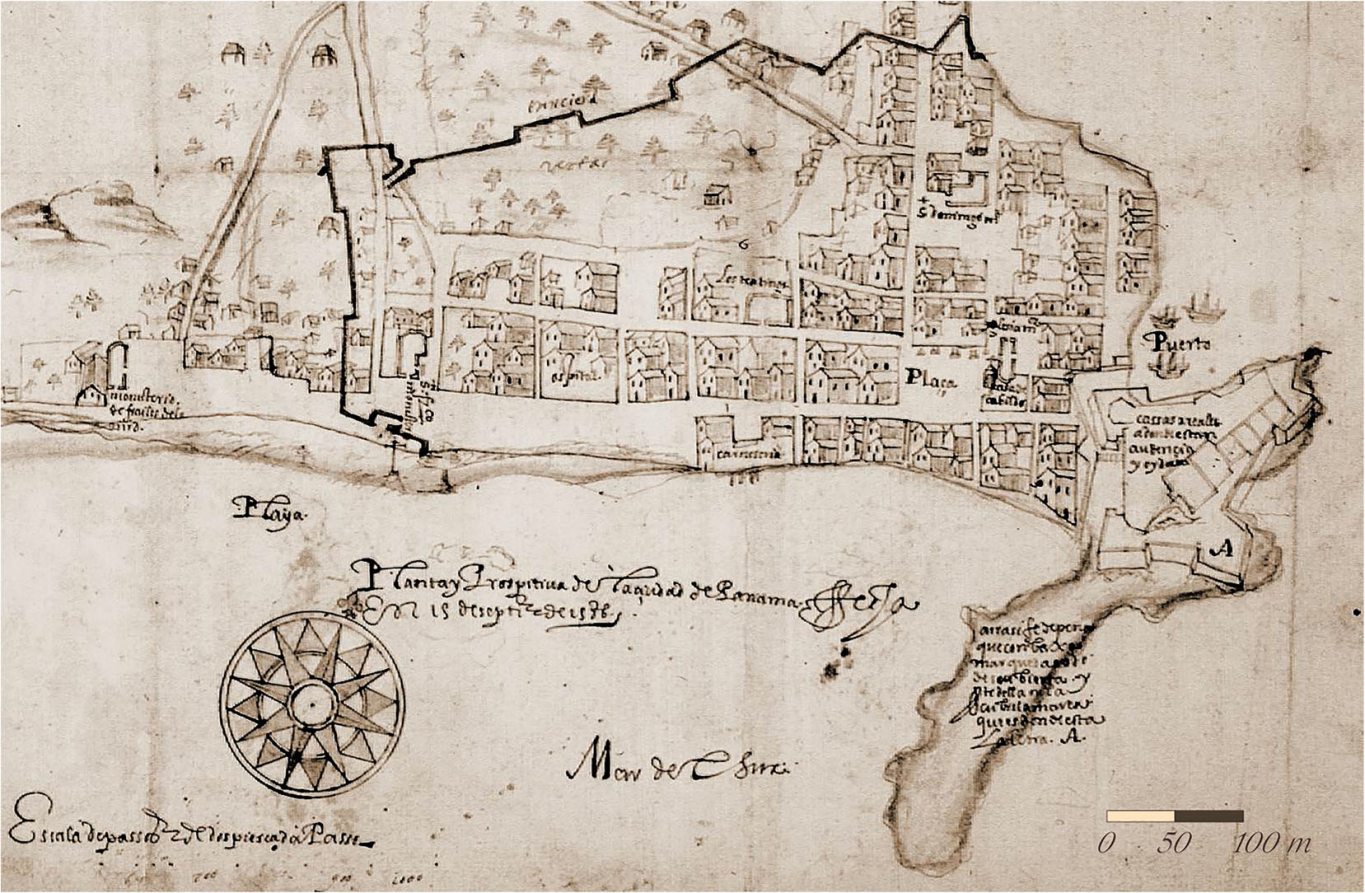
Detail of the “Planta y prospectiva de la ciudad de Panamá,” by Bautista Antonelli, 15 September 1586 (Antonelli 1586). (Image courtesy of the Archivo del Museo Naval, Madrid, Spain.)

The historical and archaeological evidence recovered establishes the site of Panama’s original principal church on the city’s main plaza and points to a foundational plan established in 1519, adjusted in response to fire, population growth, and the need to facilitate future construction in stone. These findings explain the slight irregularity in Panamá Viejo’s orthogonal design illustrated by the royal engineer, Bautista Antonelli (1586) (Fig. 2), as well as his nephew and successor, Cristóbal de Roda (Roda Antonelli 1609), and noted by architectural historians (Tejeira 1996).

## Historical Background

The founding of cities has long been recognized as essential to the Spanish conquest and colonization of the Americas (Mena García 1992; Kagan and Marías 2000; Gutiérrez 2010). Recent studies, moreover, have emphasized the mobility of some 200 urban settlements over the course of three centuries (Musset 2011:137) in response to natural disasters, defensive needs, acquired experiences, and jurisdictional claims (Lucena Giraldo 2006; Díaz Ceballos 2017). At the same time, more precise information about specific early Spanish American settlements continues to be unearthed. The fact that many early foundings proved part of a process of trial and error, however, should not overshadow recognition of other, more enduring settlements resulting from such processes, such as Panamá Viejo.

A recent contribution to the 500-year commemoration of the founding of Vera Cruz, Mexico, has dispelled confusion about the location of its first settlement on the island of San Juan de Ulúa, established on 20 June 1519 (Schwaller and Nader 2014). This foundational act entailed a legal and political stratagem that allowed Hernán Cortés to claim independence from the governor of Cuba, Diego Velázquez. By 10 July, the settlement had been moved one-half league from its original site to the mainland, with the requisite proceedings: consecration of the church on the main plaza and establishment of an urban plan following a grid scheme (Díaz del Castillo 2003:30). Five hundred years after its founding, historians have revisited the archival evidence to demonstrate that Vera Cruz underwent relocation in its first month of existence.

As for Panama, the historian Alfredo Castillero has argued for its initial foundation in the proximity of the modern neighborhood of Coco del Mar or near the Atlapa Convention Center (Castillero 2006:107–108; 2017:18), both indicated in Fig. 1, either one-half mile or one-half league from Panamá Viejo’s ruins, according to manuscript descriptions from 1607 and 1610 (Biblioteca Nacional 1607; Torres de Mendoza 1868:79–108). The first of these descriptions declared itself to be based on a report that Panama’s *audiencia* provided in response to royal questionnaires sent to all Spanish dominions. According to the *audiencia:*

Since a population seemed necessary at this place, Pedro Arias [Pedrarias Dávila] founded the city on a small hill next to certain trees that the Indians call “Panama.” The population grew with the arrival of the settlers and cathedral seat from [Santa María de] la Antigua [del Darién] and shortly thereafter moved one-half mile farther down [the coast] to enjoy the comfort of a small port [translation by the authors]. (Biblioteca Nacional 1607:60v)

The port, considered crucial in 1519, had silted up and appeared an afterthought by 1607. As a source for its information, the audiencia cited learned authorities, none of whom mentioned Panamá Viejo’s original location, as Castillero (2006) notes, before considering the testimony of “old settlers” decisive. Since no eyewitness to the events of 1519 survived in 1607, the process of information gathering relied upon and shaped old settlers’ memories.

The account of 1607 contains a number of errors. Among the most flagrant, on two occasions it confuses Vasco Núñez de Balboa, a founder and first alcalde of Santa María la Antigua del Darién, with Blasco Núñez Vela, the later viceroy of Peru. If Vasco Núñez de Balboa could be confused with Blasco Núñez Vela, might bureaucrats in Madrid compiling reports from different areas also have mixed up a more enduring settlement’s founding, such as that of Panama, with a mobile one, such as Vera Cruz? The account extracted in 1610, similar to that of 1607, corrected some errors, but incorporated others. That version alleged that Panama had been founded in 1521, not 1519, and initially at a distance of one-half league rather than one-half mile from its port. The interpretation of this location as corresponding to the Atlapa Convention Center area (Castillero 2006:107–114) (Fig. 1), gives one of Panama’s large hotels and casinos a possible, if very tenuous, claim to occupying the city’s original site. However, the testimony of multiple witnesses to Panama’s founding in 1519 in sources previously unexamined can be used to explain “old settlers’” confusion in 1607.

Panama proved as different from Vera Cruz as its founder, Pedrarias Dávila, was from Hernán Cortés. In 1513, Pedrarias had received royal instructions to establish settlements along the coast of the mainland and to oversee the trial of Vasco Núñez de Balboa, whom Cortés would imitate (Aram 2008, 2019). Specifically, the king ordered Pedrarias to establish as many settlements along the coast as necessary to maintain navigation and secure the land, selecting ports where ships could load and unload supplies to reduce the costs and burden of overland transport, and preferring healthy as opposed to swampy places, with attention to the quality of the air and water, availability of good farmland, and proximity of highlands (Ferdinand 1513). Once Pedrarias had chosen the most advantageous site possible, the king instructed him to assign lots for houses in order to lay out the settlement, with attention to “the place left for the plaza as well as that granted to the church, in addition to the arrangement of the streets [translation by the authors]” (Ferdinand 1513), which implied an orthogonal plan.

In compliance with these instructions, Pedrarias founded settlements in proximity to ports on the Caribbean coast and along the rivers of the territory optimistically termed “Golden Castile.” With the exception of Acla, however, which later incorporated Santa María la Antigua del Darién (Díaz Ceballos 2017), most of these early settlements met with indigenous opposition and failed to survive. Pedrarias also oversaw a series of reconnaissance and “pacification” campaigns. Of these, the most successful were led by his chief magistrate (*alcalde mayor*), Gaspar de Espinosa, and were designed to secure access to foodstuffs as well as indigenous labor on the Pacific coast even before founding settlements. In response to royal queries about the suitability of Panama’s location in 1534, 10 of its original founders, including Espinosa, declared that all possible options had been studied carefully before the founding of the city, including attention to the ports and rivers in compliance with the instructions that Pedrarias had received from the king. These city and church officials argued that the intervening years had confirmed the wisdom of the original location, which had been selected carefully and proved the best option (City Council and Cathedral Chapter 1534; de Andagoya 1986:219) after years of experience on the mainland. The auspicious day of the Assumption of the Virgin, 15 August 1519, was chosen for the requisite foundational ceremonies of the city baptized “Our Lady of the Assumption of Panama” (de Andagoya 1986:97).

Before Panamá Viejo’s founding, in early 1519, Gaspar de Espinosa had judged Vasco Núñez de Balboa and four of his companions guilty of treason, and Pedrarias ordered their execution. Pedrarias and Espinosa then launched a double-pronged advance that would characterize their subsequent campaigns along the Pacific coast. Pedrarias proceeded with ships and artillery to the Pearl Islands, in the Pacific Gulf of San Miguel, where the company previously led by Balboa had constructed two brigantines and was eager to explore the Southern Sea (Fig. 1). Having confirmed the men’s loyalty and appointed Gaspar de Espinosa their commander, the governor sent one of the captains who had denounced Balboa, Francisco de Garavito, to see whether Espinosa had reached the coast with another 200 men, including Francisco Pizarro, who had accompanied him overland on foot and by horse (Archivo General de Indias 1552). Men who had participated in these events testified to their experience and, at the request of Espinosa’s heirs, participated in the compilation of a record of his merits and services to the crown. Most of these witnesses had resettled in Trujillo or Lima, in Peru, some 33 years after the events that they recalled, and reported their own ages as ranging from 50 years (the case of Juan López de Aguilar) to 70 (Alonso Martín de Don Benito) and even 90 (Rogel de Loria). According to these participants’ recollections of 1519, Garavito encountered Espinosa’s party with a group of Indian fishers at Panama and rushed to convey the news to Pedrarias, who then sailed to the coast and disembarked. After surveying the area, Pedrarias and Espinosa founded the city of Panama. According to Blas de Atienza, Pedrarias and Espinosa united to found the city and to assign its first settlers lands and Indians (Archivo General de Indias 1552). Such eyewitness accounts contrast with and may also help explain the idea of the settlement’s original location one-half mile from Panamá Viejo’s main plaza, where Indian fishermen had sustained Espinosa and his troops as they awaited Pedrarias. In light of his instructions from the king and his own arrival by sea, however, Pedrarias could not fail to consider the need for a port before founding the city. Nearly one century after the event, in 1607 and 1610, the process of gathering and copying information may have led to confusion between Espinosa’s arrival in the region and Panamá Viejo’s foundation. Whatever the understanding by the 17th century, the towns and cities moved in 1519 did not include Panama.

## Archaeological Background

In the early 20th century, Panama’s National Assembly ceded its municipality the grounds and remains of the old colonial metropolis, which it recognized as national property and a public monument worthy of conservation for history and research (Asamblea Nacional de Panamá 1912). Under these social and cultural circumstances, the 400-year anniversary of the old city’s founding featured detailed attention to its principal church with emphasis on the tower’s survival alongside that of certain structures, including the lateral arch that sustained it (Lewis 1918:451,453). Juan B. Sosa undertook historical research in Seville’s Archivo General de Indias and dedicated a section of the ensuing book to Panamá Viejo’s visible cathedral, summarizing the evolution of its construction and transformations over two centuries of existence until its abandonment after 1671, as well as the subsequent reuse of its materials––including pieces of stone masonry—for the new city’s church (Sosa 1919:45–53,51).

The ruins at Panamá Viejo would not attract archaeological attention until the mid-20th century, which featured sporadic and uncoordinated incursions. Such efforts included the 1962 field season undertaken by the pioneer of historical archaeology, John Goggin, and sponsored by the University of Florida, with the goal of locating the old city’s ceramic-production kilns. Goggin studied the presence and distribution of Hispanic majolica in the Caribbean as an indicator of chronology and commercial contact, refining the typology proposed by José María Cruxent and Hale Smith (Goggin 1968:163).

In 1995 the creation of Patronato Panamá Viejo^1^ facilitated the development of a long-term plan to explore such subjects as ecological adaptation, the use of resources, and the ideological and symbolic constructions of a given period or social group based on material remains. It hoped to do so in close association with other scientific disciplines, including geophysics, archaeometry, history, and bioanthropology, while providing professional as well as specialized training in an interdisciplinary context (Rovira 2001:2; Martín and Rovira 2012:20–21; Martín and de Arango 2013; Linero and Marín 2016).

Panamá Viejo’s multicomponent complexity required fieldwork in line with the Patronato’s specific objectives, which led to a strategy of underground evaluation through prospection, permitting discoveries that would facilitate research through more extensive excavations. Moreover, this initial survey program encouraged the definition of guidelines and goals based on archaeological criteria. The surveys undertaken have sought to identify the different areas’ stratigraphic particularities, temporal relationships, and density of archaeological artifacts, as well as to define the parameters of settlements in the pre-Hispanic and colonial periods (Rovira 2001:3; Rovira and Martín 2008:17). In the case of the cathedral, all of the work undertaken from 1998 through 2005 was guided by a 1999 master plan for its conservation and preparation for visitors.

The first archaeological studies in the principal church, advanced in 1998, were intended to verify the presence of the original floor as well as the area’s stratigraphic conditions (Brizuela 1998). Further excavation of the church interior was implemented from January through March 2000, and rescue excavations took place in its atrium (Martín 2000a, 2000b). Three years later, separate rescue excavations undertaken on the southeast side of the main plaza revealed another area with colonial burials (Martín 2004). The following year, further archaeological interventions took place in the cathedral’s tower and main altar in connection with the installation of an internal stairway to reach the bell tower, with the goal of identifying, registering, and recovering the church’s features (Gómez 2005).

### Archaeology in the First Cathedral

The archaeological rescue excavation undertaken in 2003 to the southeast of Panamá Viejo’s main plaza encompassed a total area of 43 m^2^ and led to the identification of 22 colonial burials. The excavation combined stratigraphic readings with arbitrary levels based on a vertical and horizontal register from a zero-level reference point. The results indicated the area’s funerary use during the colonial period and presented elements that could contribute to interpretations of the old city’s spatial dynamics. However, at the time, Martín (2004) misinterpreted the context by assuming that the 17th-century ceramics recovered dated the deposit without considering the area’s subsequent reuse, and thereby dismissed the possibility that it could have been an early undocumented church or cemetery. Separate excavations undertaken the same year with the intention of defining Panamá Viejo’s original layout found and documented another Christian burial in front of the ruins of the Mercederian church, some 800 m up the coast from those encountered to the southeast of the main plaza.

In the units excavated in 2003, the documented burials appeared in extended dorsal *decubitus*, with the feet oriented to the east in all but one case. In general, the remains showed considerable fragmentation and deterioration due to the area’s multiple transformations, mainly from the mid-20th century. With the previous stratigraphic information, a strategy was designed to collect more precise stratigraphic and taphonomic data from the funerary contexts in Panamá Viejo’s cathedral in 2017 as well as from the southern side of its main plaza in 2018.

**Fig. 3:**
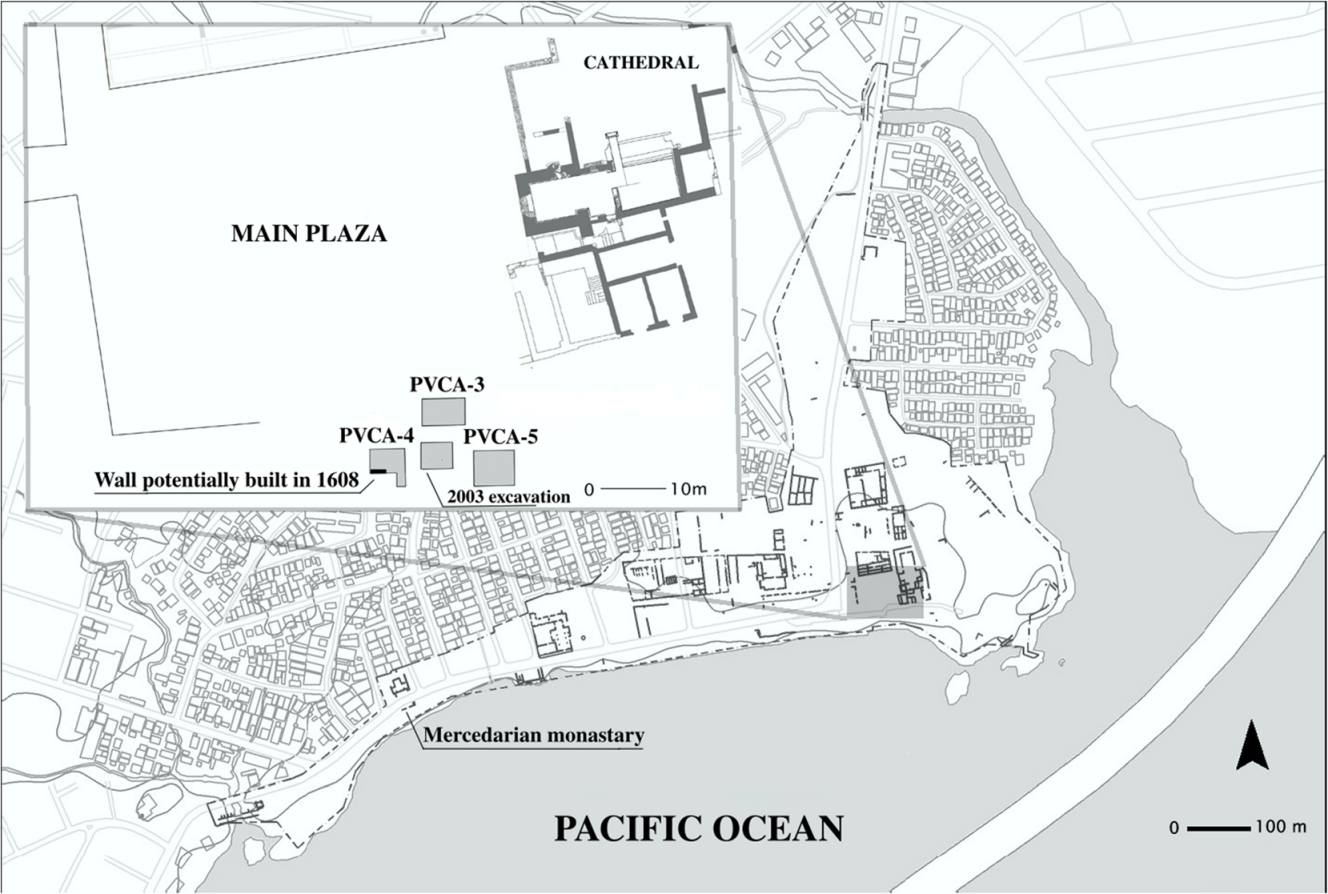
The archaeological site of Panamá Viejo: the location of the 2003 and 2018 excavations. (Drawing by Iosvany Hernández Mora, 2019.)

Excavations were carried out to the southeast of the main plaza during the dry season or first three months of 2018 (Fig. 3) in the framework of the ArtEmpire Project, with the principal investigator, Bethany Aram, as an onsite historian in order to discuss questions and finds as they emerged. Aram consulted the relevant archival documentation (previously digitized and/or online) based on the queries and observations that Juan G. Martín and Iosvany Hernández formulated as the excavations progressed. In this way, Hernández, Martín, and Aram were able to (re)interpret the fieldwork and the relevant documentation in a continual process of dialogue in order to address and refine the project’s initial hypotheses and to contrast new finds immediately and continually.

Since the early historical evidence appeared to refute the idea of Panamá Viejo’s relocation, areas chosen for intervention were situated around the funerary context encountered in 2003, with the goal of identifying its limits and possible variations in its characteristics. An excavation was planned beneath a modern street between the south of the *cabildo* and the area defined for this project, where the earliest principal church was thought to have existed in 1519–1541. The area had not been studied previously due to the presence of concrete pavement and stones, the removal of which required heavy machinery to open an excavation 5 m wide north–south, and 6 m wide east–west. Within these limits, two trenches were dug with the intention of establishing the depth of the sandy coastal strata and revealing any possible deeper pre-Hispanic features. The results of these surveys were negative, and the northern side of the block to the south of the main plaza could not be identified precisely.

Subsequently, the team decided to excavate to the west of the 2003 excavations. The survey led to identification of a funerary feature in a sand substrate some 40 cm beneath the surface level, and the area was extended to 49 m^2^. Methodologically, we applied the principles of Edward Harris, based on the stratigraphic unit (UE) as the minimal entity and registry of each level without need for horizontal markers (Harris 1991:121–145) and careful attention to horizontal associations as well as vertical relations in order to detail the different planimetric sections (Carandini 1997:66–70; Roskams 2001:186–202). Each burial was registered in relation to the inference of its sections or interfaces in order to understand the configuration of the ground on which the body was deposited, its integration in the context of funerary activities, and taphonomic aspects (Tiesler 1993:15; Duday 1997:116; Knüsel and Robb 2016:656).

Identification of the different moments of inhumation in the same area, following the arguments of Harris (1991:100), required analysis of the vertical relations produced by heterogeneous actions at different times. The relative chronology was established based on the sequence itself with support from historical documentation and archaeologically recovered artifacts.

**Fig. 4:**
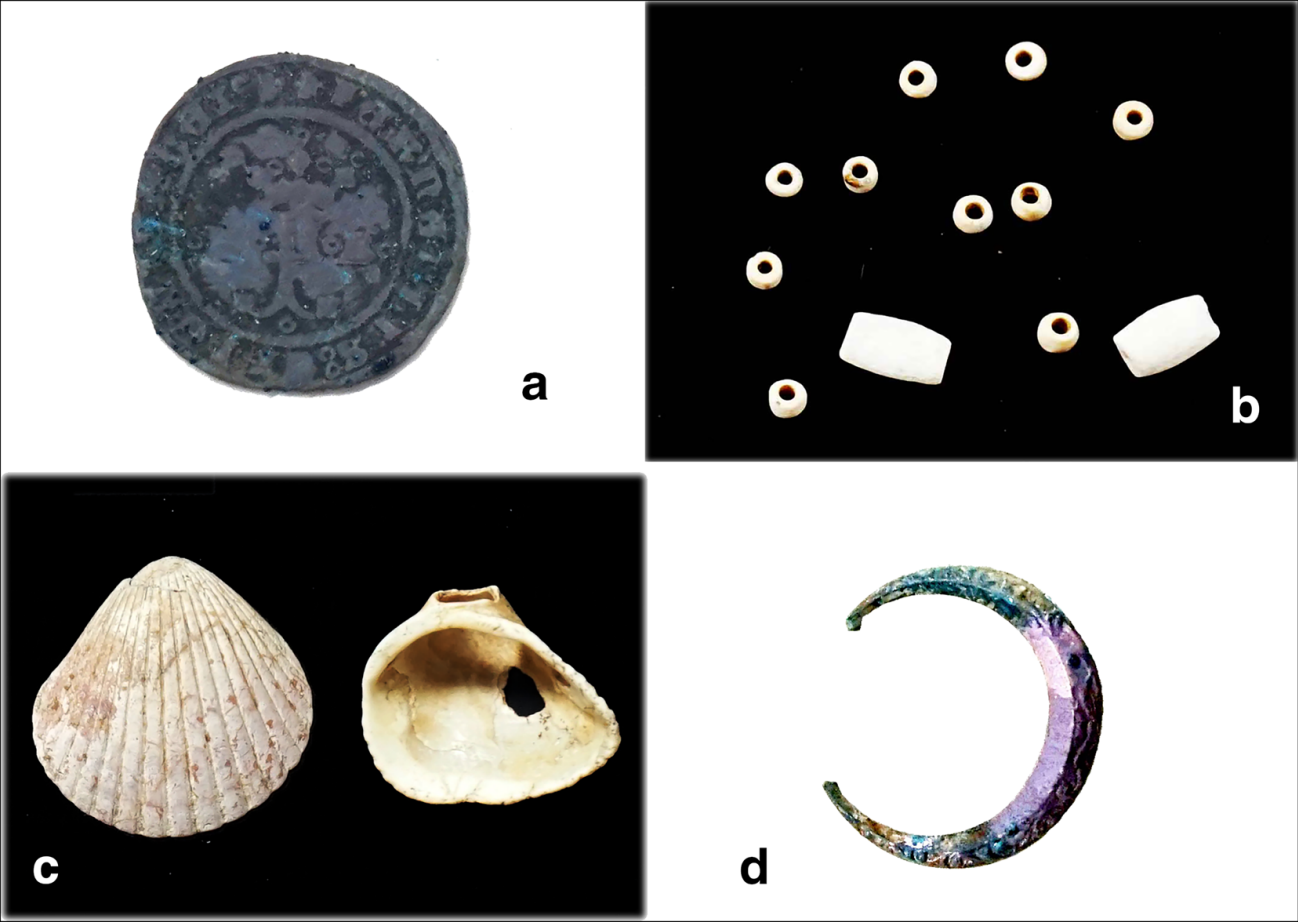
(*a*) A 2 *maravedí* silver- and copper-alloy coin minted for Santo Domingo between 1505 and 1531 (Proyecto Arqueológico Panamá Viejo [PAPV] 3/Sur de la Plaza 2018–PVCA 5, UE 4010), (*b*) beads from a 10-bead rosary made from seashells, (*c*) worked shells (*Argopecten* sp*.* and *Noetia* sp.), and (*d*) decorated clasp with a simple, D-shaped oval structure (PAPV 3/Sur de la Plaza 2018–PVCA 4, UE 4008). (Photos by Iosvany Hernández Mora, 2018.)

All of the modern structures, including the concrete floor, were documented and removed during the excavation process. In the process of this task, a silver coin minted between 1505 and 1531 was recovered (Fig. 4*a*). Beneath the modern structures there emerged a substrate that was darker, possibly due to a greater amount of organic matter. An area with a colonial floor composed of small stones joined with a mortar of sand and calcite was uncovered. In the southeast sector a piece of the base of a wall made of stones in similar mortar, 1 m wide from north to south and 1.7 m long from east to west, was also found.

**Fig. 5:**
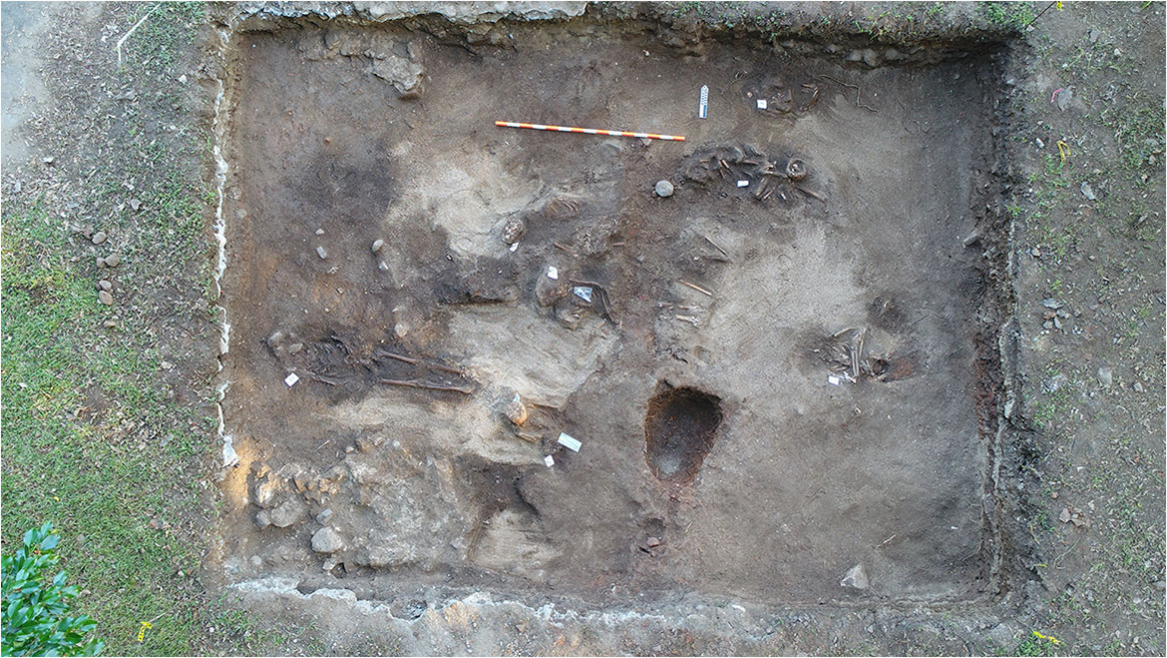
Burials inside Panamá Viejo’s first cathedral (PVCA-4). (Photo by Juan Guillermo Martín, 2018.)

The burials’ surfaces were concentrated between 39 and 49 cm deep, with a total of 10 primary interments and 2 secondary burials documented. In no case was it possible to observe the outlines of the burials, which were inferred by the system of levels, indicating concave interfaces in the most complete burials. The inhumations appeared in a sandy, very little compacted sediment, which complicated observation of the burials’ outlines or imprints (Fig. 5). The scarce material culture recovered on this level corresponds to funerary practices and religious activities of the early colonial period: stone and ceramic candelabras, fashioned seashells, pins, metalwork, a button, and rosary beads made from seashells. Various modern interventions, including the installation of tubes and electric cables, as well as the extension of the coastline toward the south for the construction of infrastructure, had altered the funerary strata.

In sum, the data obtained from the southeast of the main plaza made it possible to define an extensive and coherent area of funerary and religious activity. The burials and associated material culture excavated beneath and in proximity to the colonial floor provided a coherent view of an early funerary context largely disturbed by subsequent interventions. These findings presuppose the existence of a structure made of perishable materials that encompassed this area. Specifically, the burials studied in the units excavated in 2003 and west of them in 2018 defined a surface of approximately 67 m^2^ parallel to the southern coast of the sandy beach. The most likely explanation, in our view, is that this area represents the location of Panamá Viejo’s first cathedral.

**Fig. 6:**
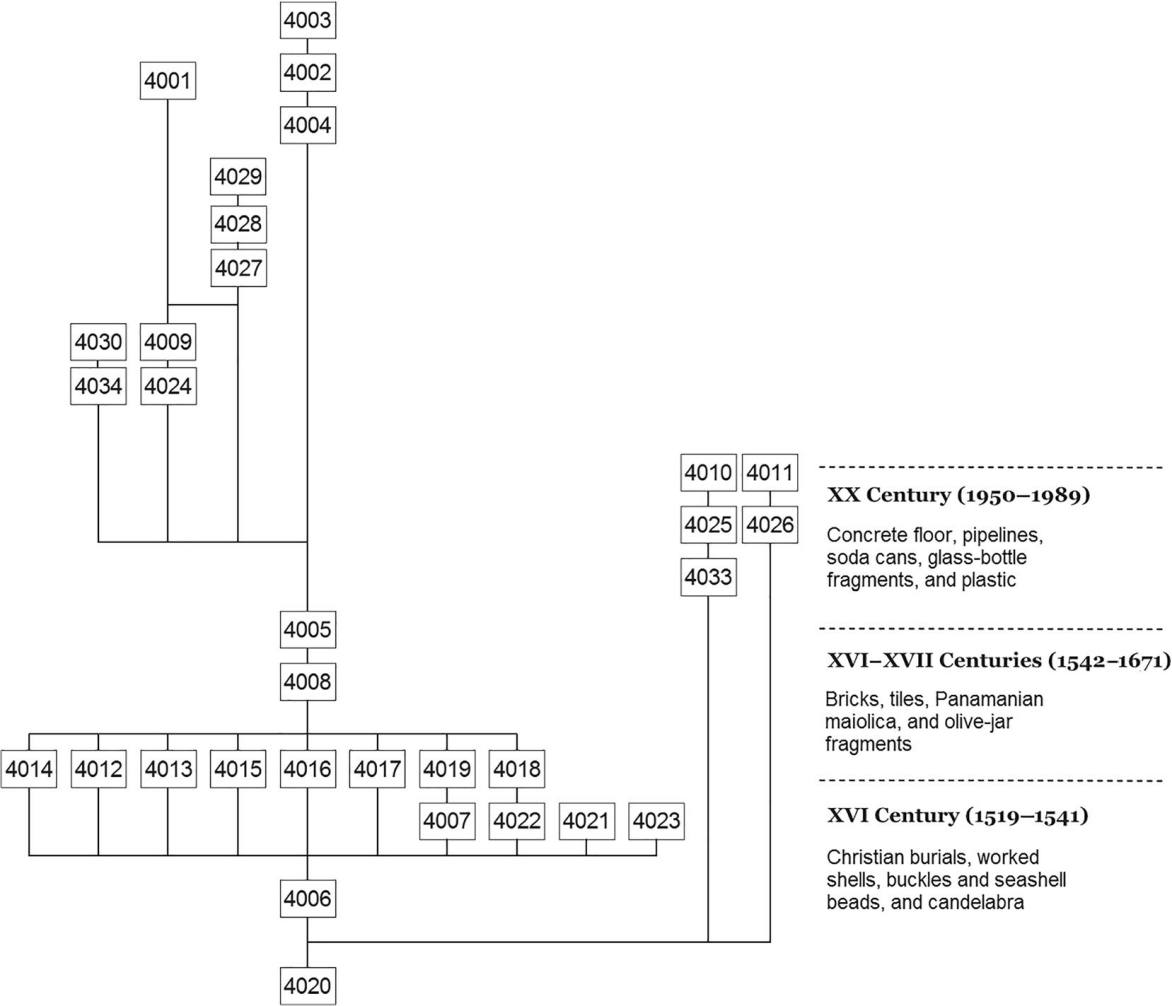
Stratigraphic sequence of Unit 4 southeast of the main plaza, showing three moments of its formation and transformation: (1) The early 16th century with burials, (2) constructive transformations in the 17th century, and (3) alterations observed in the 20th century. (Figure by Iosvany Hernández Mora, 2019.)

The stratigraphic sequence revealed the formation of the archaeological deposit to the south of the main plaza in three well-defined phases, the first in the 16th century (1519–1541), containing primary and secondary interments from the area’s funerary and religious use. A second phase identified extended from the mid-16th century until the city’s abandonment (1542–1671), when the area was used for the construction of houses, evidenced by the stone floor and foundation. Finally, a third phase encompasses the 20th-century interventions, refilling, and modifications that produced a mixture of artifactual material from the contemporary and colonial periods in the earlier levels of the archaeological context (Fig. 6).

### Toward an Integrated Interpretation of New Evidence

To illuminate the first and second periods identified, historical evidence suggests that the original plan for the city of Panama established in 1519 underwent modifications after fires in 1538 and 1540 (phase 1) and subsequently in response to a receding coastline (phase 2). Our interpretation of this data draws upon archaeological evidence of what can be interpreted as the original sites of Panamá Viejo’s two oldest churches, the principal church, or cathedral, and the Monastery of Mercedarian Friars. Historical documentation from the second quarter of the 16th century describes the rebuilding of both churches as well as other activities that subverted the city’s original orthogonal plan.

Pedrarias Dávila’s personal correspondence records his attention to urban planning in Panama and Natá, and particularly to the construction of their principal churches as well as his support for the Mercedarians. It also suggests that in 1519 Christians had raised their own makeshift church to the south of the main plaza using local materials and labor, rather than occupying a previous indigenous structure, such as those that left apparent post molds in the western Parque Morelos area of Panamá Viejo (Martín 2002). When a hurricane destroyed Panamá Viejo’s main church as well as several houses in 1521, Pedrarias oversaw the church’s reconstruction and wrote his spouse about it (Dávila 1522). With the main church rebuilt and a trail opened between Panama and Nombre de Dios, Pedrarias and Espinosa would repeat the strategy of a two-pronged advance that preceded Panama’s founding by sea (Pedrarias) and land (Espinosa) in order to establish the settlement of Natá on the Pacific coast to the west of Panama. From Natá in 1526, Pedrarias informed the provincial head of the Mercedarian order, his friend Francisco de Bobadilla, that, before leaving Panama, he had ordered a second hut built for the Mercedarians on the plot assigned to them, marked the place where it should be built, and left the wood, reeds, and straw (“madera y caña y paja”) necessary to raise it there. Upon returning from Nicaragua, Pedrarias promised that “his” natives and one of his servants, Hernán Gómez, would complete the structure (Dávila 1526). Plausibly, this hut would have been contiguous to another in which the individual found to the south of the presently visible Mercedarian ruins may already have been buried, all within the plot Pedrarias had assigned the order in 1519. Second only to a church, the friars would have needed a residence. The historical documents that survive from this early stage of settlement, like the governor’s letter, depict the use of readily available construction materials—wood, reeds, and straw—that leave scarce evidence in the archaeological record, even for the most important structures like the principal churches and monasteries.

Construction in perishable materials reflected a number of factors, including many male settlers’ preference for mobility. In 1519 Pedrarias granted soldiers reluctant to reside in Panama the incentive of natives obliged to provide service on *encomiendas*, ostensibly in exchange for Christianization, as well as a series of other privileges and tax exemptions that the king confirmed (Aram 2008, 2019). The conquest of Peru in the 1530s siphoned population from Panamá Viejo, but subsequently proved a boon for its commercial and service sectors. By the late 1530s merchants replaced adventurers at the apex of the elite strategically situated between Callao (Peru) and Seville. The indigenous population’s demographic decline also made the city increasingly dependent on free and enslaved Africans, who quickly became a majority.

In the judicial proceedings that followed a rebellion of enslaved and free blacks in 1535, one of the men involved, Juan Marinero, declared under torture that the rebels had planned to set fire to Panama’s cathedral on a Sunday, when the urban population gathered inside it (Marinero 1535). Panama’s residents were well aware that construction in wood, reeds, and straw increased even the most important buildings’ vulnerability to fire. The much-feared devastating fires, however, began in other buildings before reaching the cathedral in 1538 and, especially, 1540. According to the royal magistrate, Dr. Francisco Pérez de Robles (1540), a fire that broke out on 15 February 1540 destroyed the principal church, the bishop’s house, the city hall (although still under construction), and all of the city’s best houses and stores along the Calle de la Playa (Street of the Beach)—a road that would disappear by 1586 (Fig. 2). To reduce vulnerability to fire in the future, Robles indicated his intention to limit the use of straw in reconstruction despite the settlers’ likely resistance:

Due to the difficulty of building in stone and roof tiles, they will return to straw where they can. We will limit what they can build, since they are confined to the north [due to a river and swamps], and will not be given licenses to build with straw beyond what we can permit, so that need and narrowness [of the site] will force them to build in the Spanish way [translation by the authors]. (Pérez de Robles 1540)

Dr. Pérez de Robles’ report referred to the “narrowness” of the site as well as to a road along the beach in the most prestigious part of the city. Two years earlier, a city councilor had protested the extension of houses beyond the plots initially assigned to them and into the public streets. In particular, he protested the construction of houses along the coast, which ruined the view, accumulated trash, increased the risk of fire, and impeded passage along the beach (de Guiso 1538). By the time of Antonelli’s illustration in 1586, the Calle de la Playa, which would have been crucial to the city’s original grid design, had disappeared.

Concerns about the confined circumstances that would affect rebuilding not only pointed to the risk of fire, but also to that of floods along the coastline. Intensified human activity, including the use of mangrove vegetation in local construction (Biblioteca Nacional 1607:55v), would have precipitated and exacerbated erosion, the loss of the coastline, and silting-up of the contiguous port, popularly known as the “Tasca.” By the late 16th century the situation forced oceangoing vessels, which had increased in tonnage, to dock on the nearby island of Perico. The loss of the red mangrove tree and other vegetation along the coast also led to the disappearance of bivalves and gastropods supported by the mangrove environment and consumed in the pre-Hispanic period (Martín and Rodríguez 2006).

Erosion of Panamá Viejo’s coastline continued to the point that the ocean claimed streets, kitchens, and slave quarters, and in August 1608 the city’s best houses faced the threat of serious damage from flooding. In light of the urgent situation, the municipal council pledged 1,000 ducats toward the repairs, the additional costs of which would be divided among the owners of houses along the coast that would be protected by the wall built to retain the ocean. The budgets prepared by different masons included the construction with stones and calcite of a retaining wall 2 *varas* (1.7 m) wide (Archivo General de Indias 1608), the base of which could be that encountered beneath the colonial floor removed in the 2018 excavations (Fig. 3). This wall, if correctly identified, would have marked Panamá Viejo’s southern limit in 1608, when the ocean would have reclaimed not only the “Street of the Beach,” but also part of the site of the original cathedral.

Although fires and floods increased awareness of the advantages of building in stone, the use of wood remained common in Panama for a number of reasons. The region had experienced a precocious development of shipbuilding, to the extent that the carpenters constituted the cathedral’s oldest confraternity (Archivo Arzobispal de Lima 1583). An important sector of the population, enslaved as well as free laborers, cut, hauled, and worked with wood. The abundance of wood in the region and the importance of shipping promoted carpentry.

Even before the fires of 1538 and 1540, Bishop Tomás de Berlanga and the cathedral chapter argued for the need to rebuild Panama’s cathedral in stone. As one of the founders of Santo Domingo’s Dominican convent and its prior after 1517, Berlanga had witnessed the construction of the Americas’ first stone cathedral and probably met the master builder Antón García in Santo Domingo at the time (Palm 1955[1]:80–82; Figueras 2010). En route to occupy the bishopric of Panama in 1534, Berlanga engaged García to oversee the construction of the cathedral that he envisioned for the mainland, probably based on that of Santo Domingo, and secured royal license for García to travel to Panama with his wife and property (Dean and Cathedral Chapter 1535). The bishop repeatedly informed the king about the need to rebuild Panama’s cathedral in stone and, probably following García’s advice, defended the case for doing so slightly removed from the sandy coastline. On 15 April 1540, King Charles accepted the bishop’s argument and proposal to change the cathedral’s location:

That at the time when this city of Panama was founded (*trazado*), the cathedral church was built where it had poor visibility, and that it could be located in the forefront of the plaza where it would be more visible and honor and greatly adorn the plaza and city. And this could be done now, at the same time that it is constructed in stone, with the city giving a plot that it had for the city hall ... to the said church and receiving another plot elsewhere [translation by the authors]. (Charles V 1540a)

The bishop had argued successfully for the need to rebuild the cathedral in stone and to move its location from one side of the main plaza to another that had initially been reserved for the city hall, or *cabildo*. The elevated, rocky terrain to the east of the plaza appeared more suitable for a stone structure that would require more solid foundations than the sandy coastline could provide. Berlanga’s moral authority appears to have facilitated acceptance of the cathedral’s transfer to a rocky, north–south plot whose elevation appeared more important, symbolically and practically, than its orientation. In fact, of the many churches built in Panamá Viejo after 1541, only that of the Conceptionists achieved an eastward orientation. Even with the cathedral’s main altar to the south, the heads of the deceased buried there could (and did) face east. Finally, the shift from a pristine, austere religiosity to baroque magnificence foreshadowed the decision to relocate the principal church and can also be observed in the material culture (seashells vs. devotional medals and braided silver knots) associated with the burials in each cathedral.

Berlanga obtained extensive support from the Crown to build Panama’s cathedral in stone, but failed to see the project achieved. Not only had the king agreed to the new location, but he also pledged to pay the salaries of four qualified officials who could “teach the blacks and Indians in this province” to work in stone, while providing an additional 300,000 pesos for construction, which were finally used to purchase slaves (initially 12, but ultimately 20), who would be transported free of charge in order to build the cathedral (Charles V 1540b). Yet the ships that conveyed the royal provisions and some 200 individuals from Iberia to the Caribbean hit a reef and sank off the coast of Acla, Panama. Berlanga particularly lamented the death of several relatives and a slave he had purchased for 250 ducats, “one of the best builders in Seville” (Berlanga 1541).

This shipwreck prevented the bishop from seeing Panama’s cathedral built in stone. He did, however, oversee its relocation to facilitate future masonry. After Berlanga had returned to Castile, a witness on behalf of the provisor of the main church credited him with the transformation of what had been a decrepit hut of straw (“buhío de paja maltratada”) into a structure with 140 ft. of sturdy wood and tiles (“de madera recia y cubierto de teja”) (Archivo General de Indias 1544). By 1578, however, members of the cathedral chapter emphasized the church’s decrepit situation, ability to accommodate only one-third of the city’s population, and vulnerability to fire, corsairs, and maroons (Dean and Cathedral Chapter 1578). Such claims persuaded the king to grant funds to support the cathedral’s construction in stone, plans that advanced only after 1618. The cathedral’s uncanonical south–north orientation, while also noted in 1578, appeared an inevitable adjustment to the terrain and resources available.

Another of Panama’s first churches, the Mercedarian monastery, also appears to have changed its orientation upon moving to higher, more stable terrain farther from the coast within the plot initially assigned to it. This potential shift is indicated by the Christian burial facing east and encountered some 50 m south of today’s visible ruins with a north–south orientation (Yanaida 2003). Limited rescue excavations undertaken to recover the original urban layout in 2003 uncovered an area of stone pavement from the colonial period as well as a Christian burial positioned east–west in an area that that pavement did not cover. The orientation of this colonial burial, probably accompanied by others under the pavement, suggests that the original Mercedarian church may have shared the same canonical, eastward orientation as the first cathedral. In 1540, challenges to the Mercedarians’ ownership of the land led them to defend their rights to the plot dating from the city’s founding (Charles V 1540c). As was the case with the cathedral, the Mercederian church apparently relocated to higher ground in order to facilitate stone construction and changed its orientation from east to north in the process.

The original location of Panama’s principal church lies, literally, at the heart of its founding and is therefore crucial for ascertaining the city’s initial layout. According to historical documentation, the original structure had been destroyed twice by fire and once by a hurricane. Not surprisingly, its ephemeral foundations left no visible trace in an area heavily reused. On the other hand, human remains encountered in 2003 (22 individuals) and recovered systematically in 2018 (16 additional individuals) clearly mark the original site of the principal church. These primary burials were found in the canonical east–west orientation, most of them facing what would have been the main altar and the rising sun, with only one clear exception aligned west–east, perhaps a member of the clergy facing the congregation. In 1540 the task of building another cathedral, whether in stone or wood, must have seemed daunting enough to preclude any consideration of transferring the individuals interred in the original church to the new cathedral. Thus the dead were left behind after 1541, as they would be with the construction of another city with yet another cathedral built after 1671 in the area known today as the “Casco Viejo” (Fig. 1). Unlike their early colonial successors, however, Panamá Viejo’s pre-Hispanic inhabitants moved and reburied their deceased (Cooke et al. 2019:64–94).

### Mortuary Artifacts

Compared with pre-Hispanic or even 17th-century colonial funerary contexts, the inhumations excavated during the 2018 field season contained very sparse material related to the use of shrouds, clothing, and personal ornamentation. The even more limited number of such artifacts associated with specific burials included 12 beads found among the lowest ribs on the left side of one individual’s thorax, beads perhaps formerly held in her right hand. The beads recovered were white and made from seashells, 10 of them in a very small, cut tubular form, with a length and diameter of 0.3 cm and a hole diameter of 0.1 cm (Fig. 4*b*). The two larger beads, with the same tubular shape, were narrower in diameter at the ends like the barrel (*barril*) form Deagan (1987:161) identifies, measuring 1.1. cm long, 0.1 cm in diameter in the middle, and 0.6 cm in diameter at the ends with an hole diameter of 0.1 cm. The number of beads and their place in the grave provide evidence of their fabrication and use as a 10-piece rosary, common in the 16th century, with 10 beads used as a reminder to repeat prayers until the completion of one rosary or group of mysteries. Deagan (2002:66) describes large beads with devotional medals or crosses at the extremes. In this case, the larger beads must have finished the piece in the manner of Stations of the Cross or the Pater noster, connected with a thread of perishable, organic material. Under this grave, in another interment, two pins of copper alloy with curled tips, indicating the body’s shrouding, were encountered.

Among the artifacts recovered, a D-shaped buckle with a simple oval structure was found in a grave at the northeastern part of the excavation (Fig. 4*d*). Its diameter measured 2.9 cm at the external, rounded portion and 2.6 cm. at the endpoints of the D, with a probable length of 1.5 cm. Made in a copper alloy, it features decorative floral motifs in relief and bears traces of gold leaf. Although the extended typology of this ornamental piece for clothing ranges from the 14th through the 18th centuries, decorated buckles have been documented in 16th-century European contexts, particularly intended as the principal part of fasteners that included belts and straps (Beltrán and Miró 2013:99). In the Americas, they have been found in early colonial contexts, such as Concepción de la Vega in the Dominican Republic, with a temporal range of 1498–1562 (Deagan 2002:182).

Artifacts not associated with specific graves include a white tubular bead with rose-colored veins, possibly made from some type of calcite, 3 cm long, 0.9 cm in diameter, and with a hole diameter of 0.3 cm, with fractures at its extremes and adhering earth due to its porous composition. Similar pieces recovered from indigenous contexts in South America dated to the period of the Spanish conquest are associated with glazed colored beads, such as Nueva Cádiz and others made from different types of stones, sea mollusks, and metals (Feinzig 2017). Four copper pins with curled heads exhibited fragmentation and alterations due to use. One button, a single piece of shell nacre made into a round shape with no decoration or point of attachment, had a diameter of 1.8 cm and was 0.8 cm thick. The button’s reverse contained a small bulge as well as signs of a truncated appendage, which indicates that it must have been covered in cloth or passementerie. Covered buttons were normally made with a wood or bone base (Beltrán and Miró 2013:191–192,194). The type of button found, roughly finished on both sides, was common in the 16th century.

Two metal tips recovered were made from a copper alloy, one of them having a slightly conical shape, measuring less than 2.2 cm long and between 0.3 and 0.2 cm thick, and a second, more deteriorated, of the same thickness and 1.9 cm long. Although there is no certainty that these metal tips were part of funerary attire, their presence, together with the low frequency of buttons, points to a specific moment when late medieval European styles reached Panamá Viejo. According to Deagan (2002:174), buttons are rare in colonial contexts before 1560, when they began to replace laces in Spain.

Among the artifacts related to religious practices, the excavations recovered, on the level of the inhumations, two locally made candelabra associated with modified seashells, one of them molded in earthenware with red slip and the other carved from stone. The earthenware candelabrum presented an irregular form due to the fragmentation of the basal edges that comprised a saucer of 3.1 cm diameter to secure the candle. The other candelabrum was made of sandstone, the rough and resistant rock used after 1580 to construct the cathedral’s tower and walls (Durán 2005:48). This candelabrum, in the form of a chalice, had burn marks on its interior walls and included a conical dish for the candle that measured 3 cm wide at its upper edge and 2.2 cm at its narrowest point.

The modified seashells may also be considered religious artifacts, in light of their intentional symbolism in this historical context. These pieces appeared in four fundamental forms: oval, round, trapezoidal, and with circular perforations at the apex achieved by techniques of fracturing and cutting by percussion and abrasion that were widely practiced by Native Americans in Panama (Dacal 1978:30–31; Mayo 2004:152–161; Lammers 2008:94–99). These artifacts’ high frequency in the strata of the inhumations indicates that people probably deposited them during the burial process and in the course of daily religious and funerary practices at the time (Fig. 4*c*).

The presence of shells in the sacred space of a Christian church simultaneously favors their interpretation as evidence of syncretism in the evangelization, religious conversion, or baptism of Panama’s natives and Africans. Seashells have very particular meanings in the Catholic world and in indigenous American cosmologies as symbols associated with water, fertility, and birth, meanings undoubtedly employed in strategies to convert the natives and to encourage them to appropriate Catholicism (Eeckhout 2004:28–29; Hernández Ramírez and Izquierdo Díaz 2014:128–129; Izquierdo Díaz and Hernández Ramírez 2017:38–42).

*Argopecten circularis* and the Spanish scallop shell, or *Pecten jacobaeus*, common on the Atlantic coast of Galicia, belong to the same family, Pectinidae, sharing similar valves and grooves formed by radial ribs marking growth, as well as ears at the terminal umbo. The scallop shell’s symbolic meaning in Catholicism, which dates back to the early Jacobean pilgrims, did not preclude its associations with fertility, the moon, and women (Sánchez and Baños 2013:65–67). Such symbolic conjunctions may have favored the introduction of *A. circularis* into the baptisms of indigenous women, although the modification of its valves and consequent distancing from the natural forms respected in the Catholic world signals an important contrast.

### Final Considerations

New evidence uncovered by archaeologists as well as historians confirms Panamá Viejo’s single location from 1519 through 1671. Findings, from Christian burials to scallop shells to reports of floods and fire, acquire significance through interdisciplinary analysis. In this way, the absence of certain structures due to their organic composition (such as the remains of the early wooden church) becomes as significant as the presence of others (such as a wall plausibly constructed against flooding in 1608). The cathedral, not the city, relocated after 1540 due to fire and coastal erosion. Indeed, fires and floods remained arbitrators of Panamá Viejo’s existence from its foundation in 1519 to its destruction in 1671. During this period, the first cathedral erected alongside the Pacific Ocean was transferred to higher ground and continually rebuilt, while remaining on the same main plaza.

Within the city founded by Pedrarias Dávila, two of the first structures built, the cathedral and the Mercedarian monastery, moved away from the coastline to facilitate their construction in stone. Judging from early colonial funeral contexts initially encountered by chance, the orientations of the churches, as reflected in the human remains buried according to Catholic rites, also shifted from an eastward to a north–south orientation. These adjustments explain deviations from strict regularity in the city layout recorded in the maps of Bautista Antonelli and Cristóbal de Roda, and visible in Panamá Viejo’s ruins today. Relocation of some of the most important structures after the fire of 1540 responded to the need for higher ground more protected from the ocean, where solid foundations could eventually sustain construction in stone.

Most notably, the “Street of the Beach” mentioned in early documents disappears from Panama’s map by 1586. Only the human remains buried according to Catholic ritual and the associated material culture mark the site of the original, principal church, which would have extended from the main plaza to the ephemeral road along the coast. Early settlements featured provisional structures, the most enduring remains of which may be those of the dead they left behind.

A map preserved at the Archivo General de Indias in Seville suggests that the principal church of another well-known archaeological site, Campeche, Mexico, underwent a similar relocation to distance the main church from the coast. The illustration, dated 1609, shows both an old church (“iglesia vieja”) as well as the “floorplan of the church that will be rebuilt on this site, with steps and a cemetery” (Archivo General de Indias 1609). Movable churches, associated with human remains, but not necessarily enduring structures, offer new approaches to at least two of Spanish America’s settlements and archaeological sites.

Our findings to the south of Panamá Viejo’s main plaza indicate that both historical and archaeological evidence can be incomplete and misinterpreted. In our study, each discipline has challenged aspects of the other’s established truth. Reconsidered together, the archaeological and historical evidence make a convincing case for Panamá Viejo’s permanence from 1519–1671. They demonstrate that the first cathedral on Pacific coast of the Americas was relocated and rebuilt according to the city inhabitants’ changing needs, values, and resources.

Unlike the region’s pre-Hispanic inhabitants, Panamá Viejo’s early colonial population shoved aside or abandoned, but did not rebury, the dead. Strikingly and fortunately for scholars, the cathedral relocated without its deceased after 1541 and again after 1671. By the mid-16th century, most participants in the city’s founding had died or moved on, leaving little recollection of the original cathedral or attachment to its burials. An early mixture of peoples and cultures emerges from the material reality left behind.

## Footnotes

1 A nonprofit organization in charge of the archaeological site’s management (Martín and de Arango 2013).
